# Long Term Cell Immune Response to COVID-19 Vaccines Assessment Using a Delayed-Type Hypersensitivity (DTH) Cutaneous Test

**DOI:** 10.3390/diagnostics12061421

**Published:** 2022-06-09

**Authors:** Yvelise Barrios, Cristina Alava-Cruz, Andres Franco, Victor Matheu

**Affiliations:** 1 Immunology Laboratory, Hospital Universitario de Canarias, 38320 San Cristobal de La Laguna, Spain; ybarpin@gobiernodecanarias.org (Y.B.); aframas@gobiernodecanarias.org (A.F.); 2 Department of Allergy, Hospital Universitario de Canarias, 38320 San Cristobal de La Laguna, Spain; calacru@gobiernodecanarias.org

**Keywords:** T-cell response, COVID-19 vaccination, DTH, skin test, SARS-CoV-2

## Abstract

Objective: As we progress with mass vaccination against SARS-CoV-2, there are key questions about the immunogenicity of COVID-19 vaccines that still are not answered. Conventional methods to measure cellular immune responses are complex and expensive in a pandemic situation. Patients and methods: Forty healthy healthcare workers accepted to participate during the vaccination schedule with a mRNA vaccine against SARS-CoV-2. Measurement of Delayed-Type Hypersensitivity (DTH) cutaneous response after intradermal test of protein S of SARS-CoV-2 at day 35 and day 200 was performed. At the same time, a specific anti-RBD IgG using a classic ELISA before vaccination, and on days 0, 35, and 200 was performed. Results: All 40 individuals had a positive DTH skin response at day 35, whereas 39 participants had a positive skin test at day 200. Moreover, although all 40 individuals showed a positive humoral response of specific IgG against spike protein at day 35, with most of them having significantly lower levels at day 200. Conclusion: DTH could be proposed as an ideal and easy method to predict cellular immunity response to mRNA vaccines 200 days after starting an immunization schedule with mRNA vaccine for COVID-19.

## 1. Introduction

Vaccination has produced a decrease in hospital admissions in intensive care units and an extraordinary decrease in deaths in the COVID-19 pandemic [1,2]. In addition, the high vaccination rates achieved in a short space of time has produced in countries like Spain an even bigger decrease in all these figures.

At the same time, the question asked of how long the immune response lasts has not yet been determined. It was found that after infection by SARS-CoV-1 the duration of the immune response could reach 2 years, but until the COVID-19 pandemic it has only been possible to verify that after infection by SARS-CoV-2 the response immunity can be more than 15 months.

Furthermore, the presence of an immune response after vaccination is something that we must assess to understand the evolution of the immune response elicited by the vaccines over time. Currently, in addition, the dilemma about the option of an additional dose of vaccine would require studying whether it is necessary to recommend it globally or whether it is necessary to individualize each case.

Knowing the state of our immune system with traditional methods of measuring cellular and humoral immunity in a pandemic situation is complex [3], expensive, and invasive, so we need simple tools that give us an immediate response to our current needs and thus distribute the doses of vaccines to those who really need them [4]. In this brief report, we tried to assess with a simple, cheap, and easy-to-interpret test, a first step to study the immune response in vaccinated individuals 6 months after the start of the immunization scheme with 2 doses of an mRNA vaccine [5].

## 2. Materials and Methods

### 2.1. Participants, Vaccines, and Schedule

The National COVID-19 Immunization Plan organized by the Health Public Plan included healthcare workers among the priority groups for vaccine administration and received the first dose (day 0) of the BNT162b2 mRNA Pfizer/BioNTech COVID19 vaccine (Comirnaty) during first months of 2021 [2]. Three weeks later the second dose of the same vaccine was administered (day 21). Our cohort studied from the beginning consisted of 40 healthy healthcare workers. All included subjects received full information and a written informed consent. Serum samples were collected before vaccination (day 0), 2 weeks after the second dose (day 35) and 200 days (more than 6 months) after the first dose of the vaccine (day 200). The study was approved by the Ethical Committee.

### 2.2. Delayed-Type Hypersensitivity (DTH) Skin Test

We performed a delayed type hypersensitivity (DTH) cutaneous test with RBD protein (CoviDCELL^®^) on the forearm at day 35 and day 200 as described previously [5]. DTH response was measured at the puncture site at 6 (T6), 12 (T12), 24 (T24), 48 (T48), and 72 (T72) hours after intradermal test (IDT), and the skin response was classified as negative (anergic: 0–1 mm) and positive (no-anergic > 2 mm) [6]. Before this IDT, all the participants had signed an informed consent [5,7]. The patients tried to provide photos by smartphone with a ruler (international metric system) placed parallel to the forearm.

### 2.3. Serology

Serum samples were collected in health worker individuals before vaccination day 0, day 35, and day 200. In some cases, an additional sample was taken 20 days after the first dose (Day 20) of the mRNA vaccine. Every sample was sent to the Immunology laboratory, and a commercial ELISA IgG specific for S1 protein of SARS-CoV-2 was used according to the manufacturer’s instructions (Euroimmun, Lübeck, Germany). Results were expressed as optical density (OD) ratios as it has been described and validated for these determinations [8]. OD ratios under 0.8 were considered negative.

### 2.4. T-Cell Response by In Vitro Stimulation with SARS-CoV-2 Spike Protein

An in-vitro diagnostic method that measures a component of cell-mediated immune reactivity to the S1 protein of SARS-CoV-2 was used. An interferon (IFN) gamma release assay (IGRA) investigated the spike-specific T-cell responses 10 weeks after the second dose of the vaccine. If the patient had been in contact with the S1 protein of SARS-CoV-2, their white blood cells would release IFN-gamma in response to contact with the antigen. The assay was performed using fresh whole blood collected from study subjects using the following method. Briefly, human lithium-heparin whole blood was obtained from each patient and 500 µL was distributed and mixed on each tube of one set of stimulation tubes (#1: blank, #2: S1 domain from the spike protein, #3: mitogen causing unspecific IFN-gamma secretion from T cells). Incubation at 37 °C for 16 h was undertaken to promote the release of interferon gamma by T cells in response to contact with the antigen. Finally, individual supernatants of these stimulated samples were collected, and an IFN-gamma ELISA (Euroimmun) was performed following the manufacturer´s indications. Calibrated standards were included and expressed in international units per milliliter (IU/mL).

## 3. Results

### 3.1. Delayed-Type Hypersensitivity (DTH) Skin Test

**Figure 1:**
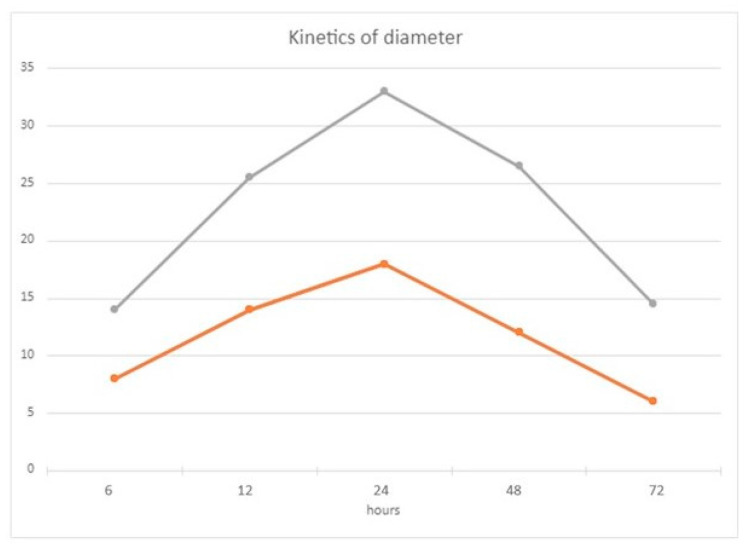
Variation of diameter (median) in subjects (compared of Diameter (median) of the DTH skin test in subjects at day 35 (grey line) and 200 (orange line)).

Skin DHT test was performed on day 35 after the second dose of mRNA vaccines; all participants (40/40) showed positive reactions. The participants were 29 females (45.6 y.o) and 11 males (48.2 y.o.). Median diameter was 8 mm (T6), 14 mm (T12h), 18 mm (T24h), 12 mm (T48h), and 6 mm (T72h). The largest diameter of the cutaneous response is obtained on average 24 h after the application of the cutaneous test (Figure 1).

**Figure 2:**
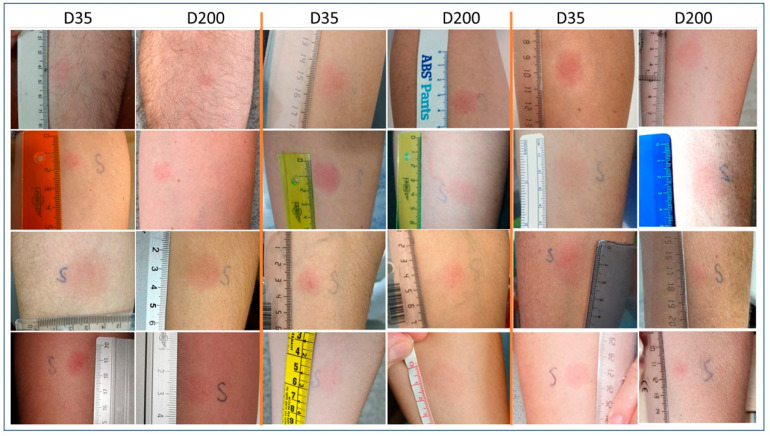
Representation of picture of DTH results from some patients at day 35 and after repeating the DTH at day 200.

After Day 200, only one subject had a clearly negative DTH response. The rest of the individuals (39/40) showed a positive skin test with a median diameter of 6 mm (T6h), 11.5 mm (T12h), 15 mm (T24h), 14.5 mm (T48h), and 8.5 mm (T72h) (Figure 1). The biggest cutaneous reaction appeared also after 24 h after the IDT puncture. The kinetic response of the skin test was similar both at 35 and 200 days after vaccination (Figure 1), with no significant differences (Figure 2).

### 3.2. Serology

**Figure 3:**
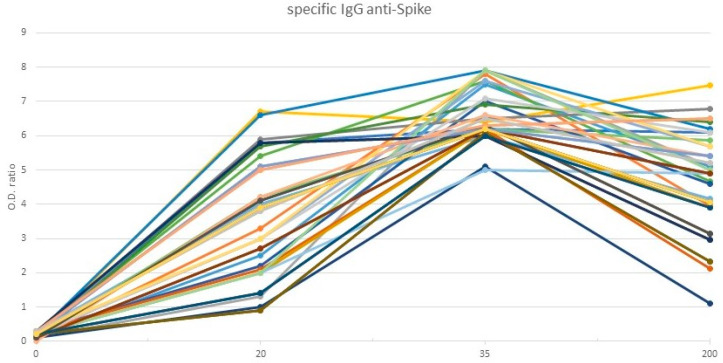
Variation of levels of specific IgG anti S at days 20, 35, and 200 in immunocompetent subjects.

All participants (40/40) maintained an immune response with positive specific IgG anti-S levels, greater than 0.8 (OD), at day 200 after the vaccination schedule started (mean 4.82 OD; median 5.05 OD), with the maximum peak being at day 35 (mean 6.64 OD; median 6.35 OD). In 30 individuals, an additional sample on day 20 was drawn, showing that the first dose had an initial impact with virtually all patients included in the positivity range (mean 3.62 OD; median 3.55 OD) (Figure 3). Only in the case of the patient who had a negative DHT test at 200 days, IgG levels decreased more strikingly than in the rest of the sample, although it was higher than 0.8 (1.1 OD).

### 3.3. IFN-Gamma Levels after Incubation of T-Cells with Spike RBD

Levels of IFN-gamma levels after specific stimulation of T cells with the spike antigen of SARS-CoV-2 were performed in the case of doubt or negativity in the DTH response. In this regard, there is just one individual in this situation. Moreover, in-vitro T cell assay showed no detectable secretion of IFN-gamma after specific incubation of T cells with spike RBD. This individual also had an IFN gamma response after unspecified stimulation of T cells that were considered positive, ruling out a T-cell general defect. The rest of the subjects showed positive IFN-gamma levels after specific stimulation with spike protein of SARS-CoV-2.

## 4. Discussion

In the previous months, various international organizations have begun to present their recommendations [9] regarding the possibility of administering a third dose to some segments of the population that are vulnerable or susceptible to severe forms if infected by SARS-CoV-2 according to antibody test response trends [10].

This recommendation would be based on the possibility that this additional dose improves the immune response capacity. In parallel, there is a scientific sector that believes it would be better to know and to study the immune response in these populations after the first setting of vaccination. Some studies have observed how the antibody titer decays over time after vaccination; moreover, cellular response studies are scarce due to the cost and the real capacity of laboratories to carry out such studies to large masses of populations.

We have studied a small sample of the population, such as the health sector, who were among the first groups to receive vaccination against COVID-19. We observed that approximately 6 months after the start of immunization, the antibody titer against RBD spike protein decays, although they remain positive in practically all the individuals. The cellular response was also maintained, except in one immunocompetent subject but with an antibody level bordering on positivity. The IgG highest value observed at day 35 was coincident with the expected maximum expression of humoral immune response after two doses of the COVID-19 vaccine [11]. This peak was also coincident with the maximum number of positive DTH-RBD cutaneous test participants (40/40) and correlates with maximum levels found in the in-vitro IGRA results in those samples that were studied.

The skin test shows that it remains a very useful tool due to its good correlation between cellular in-vivo measurement/specific IgG response along the time, although we need more studies to expand and see if with the passage of time the humoral response is maintained, and the patients do not convert to a negative skin test. Our findings support that the DTH skin test is a helpful tool to investigate the immune response to COVID-19 vaccines in a large population group given its safety, precision, simplicity, and speed of application.

With the approval of highly effective coronavirus disease 2019 (COVID-19) vaccines, functional and lasting immunity to severe acute respiratory syndrome coronavirus 2 (SARS-CoV-2) is currently under investigation as antibody levels in plasma were shown to decline during convalescence. All the vaccines approved induced a strong antibody response that has been demonstrated to last months after two doses [12]. However, the decay of antibodies elicited by COVID-19 vaccines has shown that while a significant proportion of individuals may maintain long-term titers and protection from severe infection by an antigenically similar strain, they may become susceptible to mild infection given the physiological decline of antibodies. Since the absence of antibodies does not equate to absence of immune response, it is necessary to study T cells, which have a central role in fighting off infections and, crucially, in establishing long-term immunity. COVID-19 T cell-specific immune responses have been detected at least 8 months after natural infection [13], but there are controversial results regarding T cell immunity after vaccine administration. Strategies to rapidly assess T-cell responses are therefore likely to be important for assessing immunity in the global population. Our group has validated the use of DTH as a feasible method to analyze many natural infected patients and vaccinated individuals [5,7]. In this report, we showed the results obtained in 40 immunocompetent HCW followed with this cutaneous test 200 days after receiving two doses of the Pfizer vaccine. At the same time, we also performed humoral immune and in-vitro T cell conventional methods to study the correlation among in vitro vs. in vivo cellular immune responses. The results showed that only 1 out of 40 individuals experienced a dermo-conversion of the DTH after 200 days of vaccination, whereas all of them have lower antibody levels, including the negative-DTH individual who was also negative for specific anti-IgG detection. These results support the notion that after two doses, cellular immune response measured by this in-vivo test could be an ideal biomarker to address the question of immunogenicity elicited by vaccines.

DTH has the advantage of being a simple and easy test to interpret that can be applied in hundreds of individuals without the need for any blood extraction. Moreover, in our hands, it has an excellent correlation between in-vitro vs. in-vivo results. Showing that the DTH test could be a very convenient tool to design and improve vaccine strategies like heterologous vaccination schedules. It can be also used as a first-step screening method [14] to decide on whether successive doses of vaccines are needed in vulnerable group of patients based on an objective method.

## 5. Conclusions

As we progress with mass vaccination against SARS-CoV-2, there are key questions about immunogenicity of COVID-19 vaccines that still are not answered. One of the reasons for this is the lack of suitable cellular immune methods that are easy to perform in a substantial number of vaccinated individuals. Conventional methods of measuring cellular and humoral immunity are complex and expensive in a pandemic situation. Here we present data about in-vivo DTH results on the long-term follow-up of vaccinated immunocompetent health care workers, showing that DTH could be a suitable and affordable method to understand immunogenicity elicited by COVID-19 vaccines. This tool could be used to personalize vaccine administration to optimize immune responses obtained in vaccinated individuals.

## Data Availability

Data results can be provided by request to authors.
